# Early Transmission Dynamics of Novel Coronavirus (COVID-19) in Nigeria

**DOI:** 10.3390/ijerph17093054

**Published:** 2020-04-28

**Authors:** Oyelola A. Adegboye, Adeshina I. Adekunle, Ezra Gayawan

**Affiliations:** 1 Australian Institute of Tropical Health and Medicine, James Cook University, Townsville 4811, Australia; 2 Biostatistics and Spatial Statistics Research Group, Department of Statistics, Federal University of Technology, Akure 340271, Nigeria

**Keywords:** COVID-19, coronavirus, Nigeria, travel, importation, reproduction number, Africa, infectious diseases

## Abstract

On 31 December 2019, the World Health Organization (WHO) was notified of a novel coronavirus disease in China that was later named COVID-19. On 11 March 2020, the outbreak of COVID-19 was declared a pandemic. The first instance of the virus in Nigeria was documented on 27 February 2020. This study provides a preliminary epidemiological analysis of the first 45 days of COVID-19 outbreak in Nigeria. We estimated the early transmissibility via time-varying reproduction number based on the Bayesian method that incorporates uncertainty in the distribution of serial interval (time interval between symptoms onset in an infected individual and the infector), and adjusted for disease importation. By 11 April 2020, 318 confirmed cases and 10 deaths from COVID-19 have occurred in Nigeria. At day 45, the exponential growth rate was 0.07 (95% confidence interval (CI): 0.05–0.10) with a doubling time of 9.84 days (95% CI: 7.28–15.18). Separately for imported cases (travel-related) and local cases, the doubling time was 12.88 days and 2.86 days, respectively. Furthermore, we estimated the reproduction number for each day of the outbreak using a three-weekly window while adjusting for imported cases. The estimated reproduction number was 4.98 (95% CrI: 2.65–8.41) at day 22 (19 March 2020), peaking at 5.61 (95% credible interval (CrI): 3.83–7.88) at day 25 (22 March 2020). The median reproduction number over the study period was 2.71 and the latest value on 11 April 2020, was 1.42 (95% CrI: 1.26–1.58). These 45-day estimates suggested that cases of COVID-19 in Nigeria have been remarkably lower than expected and the preparedness to detect needs to be shifted to stop local transmission.

## 1. Introduction

The novel coronavirus (COVID-19) caused by severe acute respiratory syndrome coronavirus 2 (SARS-CoV-2) emerged in the city of Wuhan, China in late December 2019 and was declared a global pandemic by the World Health Organization (WHO) on 11 March 2020 [1]. In the time since, the disease has quickly spread to all continents and, to date, over 1.6 million cases have been recorded with a fatality rate of 6.19% noted on 11 April 2020 [2].

Thus far, the risk of COVID-19 importation from Europe to Africa is higher than the risk of importation from China [3]. In their study, Martinez-Alvarez et al. [4] compared early transmission of COVID-19 (within 6 days after the first cases were detected) in selected countries and observed a more rapid spread of the virus in some West African countries than in Europe [4]. The situation in African countries could be worse than what is being reported, as most of the countries are inadequately prepared for disease outbreak due to poor disease surveillance and response systems, as well as inadequate and overstretched health facilities and services. However, African countries with the highest importation risk have also been found to possess a high capacity to respond to outbreaks [5]. As of 11 April 2020, a total of 13,814 confirmed cases and 747 deaths from COVID-19 have been documented in Africa [2].

Although the first case of COVID-19 in Nigeria was detected on 27 February 2020, this did not lead to an immediate outbreak. The epidemic trajectory has been slow, in part, due to the public health interventions implemented in Nigeria, which reduced both local transmission and importation [6,7]. A series of immediate interventions were put in place by the government of Nigeria in response to COVID-19. Among others, an immediate international travel ban was imposed on 15 countries on 20 March 2020, and all schools and universities were closed in order to minimise mass gatherings. 

There are many epidemiological characteristics of the early dynamics of COVID-19 for different countries where there was an outbreak, but none for Nigeria. In this article, a preliminary epidemiological analysis of the first 45 days of COVID-19 outbreak in Nigeria is provided. With increasing importation of COVID-19 into Nigeria, a large disease outbreak is imminent, as consistent with observed cases in countries that are epicentres. One key variable for measuring transmissibility of infectious diseases is the effective reproduction number ($R_{eff}),$ which is related to the basic reproduction number ($R_{0})$. The basic reproduction number ($R_{0})$ is the average number of secondary cases that arises when one primary case is introduced into an uninfected population [8]. It is called the effective reproduction number, ($R_{eff})$ when this value changes during an epidemic [9]. Travel has remained a major source of concern for the current COVID-19 pandemic; therefore, early transmissibility of the disease in Nigeria was quantified using a sequential Bayesian method, adjusting for disease importation.

## 2. Methods

In this study, the daily number of confirmed cases of COVID-19 were obtained from publicly available outbreak situation report of the Nigeria Centre for Disease Control (NCDC) [7] and the World Health Organization daily situation reports [2].

The real-time growth of COVID-19 in the first 45 days was estimated by fitting exponential curves to the daily counts and its changes in time, based on the log-linear Poisson regression model. Transmissibility of the disease, measured by the effective reproduction number ($R_{eff}$), was estimated from the epidemic curve. To account for the effect of disease importation, a sequential Bayesian method was used to estimate time-varying $R_{eff}$ from the incidence series with a sliding window [10]. Previous studies have estimated the mean serial interval (time interval between symptoms onset in an infected individual and the infector) of COVID-19 to be 7.0 (95% CI: 5.8–8.1) days, with a standard deviation of 4.5 (95% CI: 3.5–5.5) days [11]. Therefore, a shifted gamma distribution with shape and scale parameters taken as 9.8 and 0.7, respectively (corresponding to a mean of 7.0 days and standard deviation of 4.5 days) with a shift of 1 day was assumed and used for estimating the distribution of the serial interval. 

A sensitivity analysis was carried out to investigate the effect of changes in the sliding window on cases $R_{eff}$. All analyses were done in R software version 3.6.2 [12] using packages incidence [13] and EpiEstim [10].

## 3. Results

**Figure 1:**
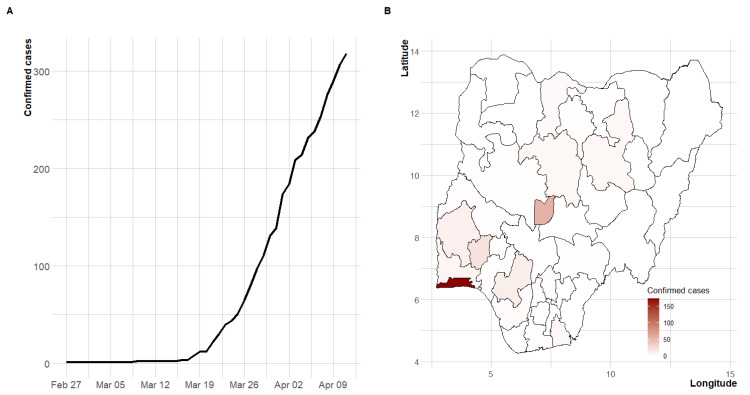
Distribution of COVID-19 disease in Nigeria between 27 February 2020 and 11 April 2020. (**A**) Time series plot of daily counts, (**B**) states affected by the disease.

**Figure A1:**
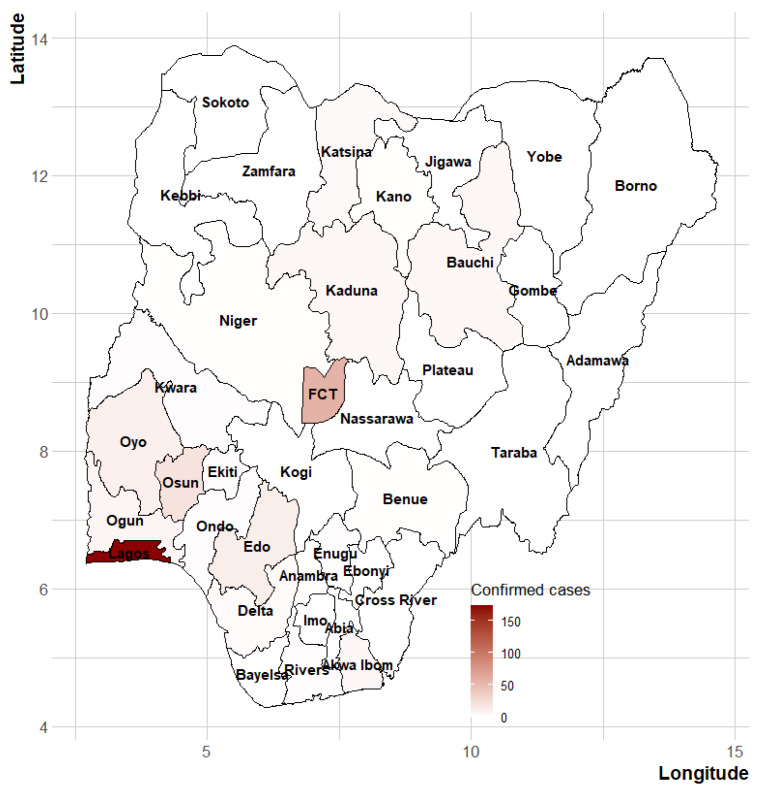
Map of Nigeria showing the distribution of cases of COVID-19 as at 11 April 2020.

**Figure A2:**
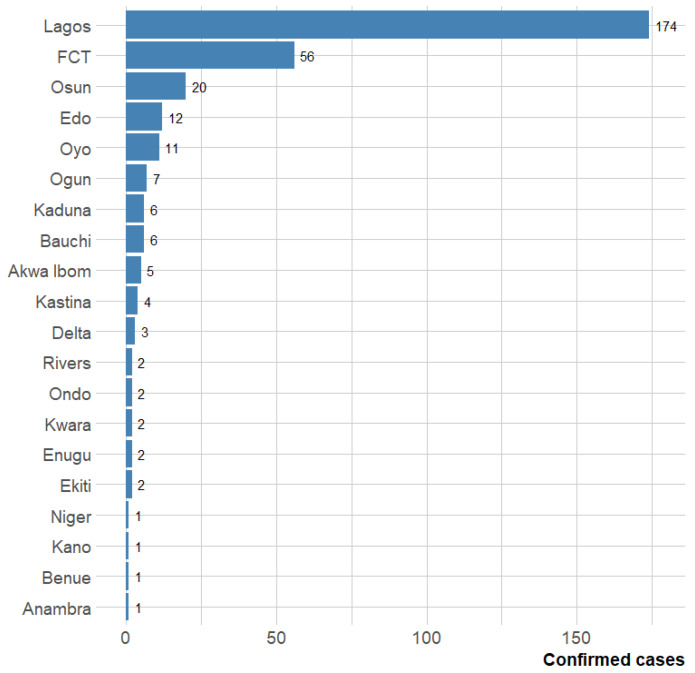
The number of confirmed cases per states in Nigeria.

Between 27 February 2020, and 11 April 2020 (a 45-day period), a total of 318 confirmed cases and ten COVID-19 deaths (case fatality rate of 3.14%) were recorded in Nigeria. Figure 1 presents the cumulative number of confirmed cases over time and the geographical coverage of the disease. The temporal trend of the incidence showed an exponential growth. The first case of COVID-19 occurred in Lagos state, the economic hub of the country, which has remained the focus of the pandemic in Nigeria. About 72% of the cases were reported from the duo of Lagos and the Federal Capital Territory (FCT). More than 47% of the cases are imported cases (travel-related) and the spread of the disease has been concentrated in the southern region of the country and the FCT (Figure 1, Figure A1 and Figure A2). 

**Figure 2:**
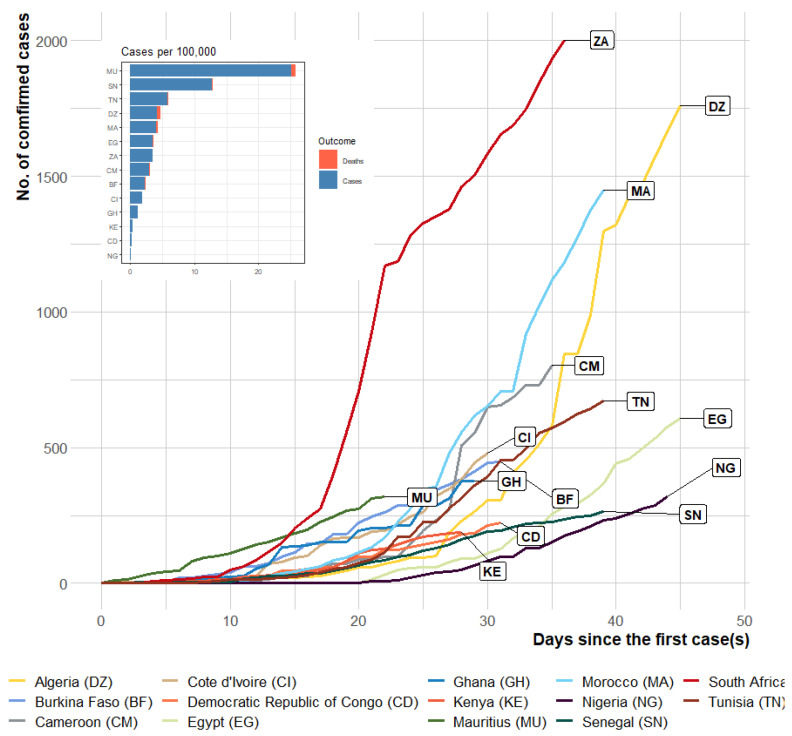
The number of confirmed cases in the first 45 days of COVID-19 importation to selected African countries. Inset: cases per 100,000 population as of 11 April 2020.

**Figure 3:**
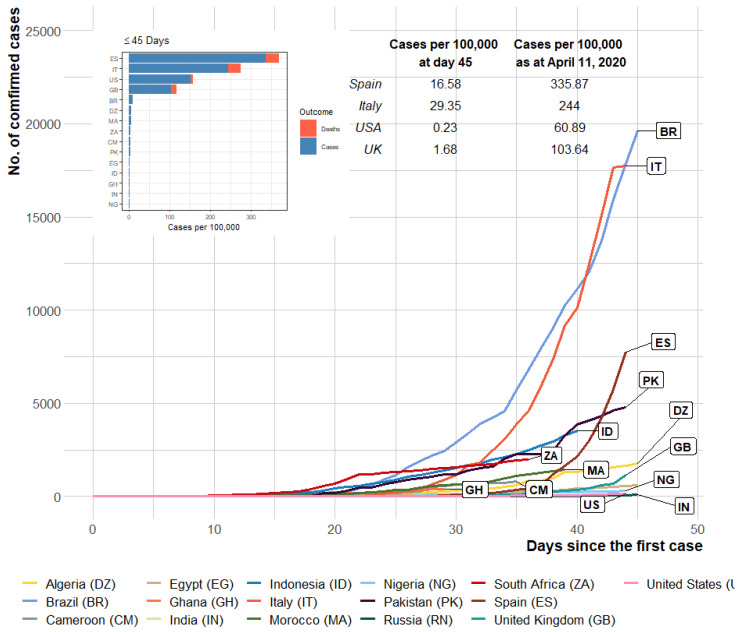
The number of cases in the first 45 days of COVID-19 arrival to selected countries (inside and outside Africa). Inset: bar plot showing cases per 100,000 population at day 45. Comparison of cases per 100,000 on day 45 and on 11 April 2020 (around a month later).

For comparison, the observed daily cases in selected African countries from day 1 up to day 45 are shown in Figure 2 and Figure 3. Initial inspection of the progression indicates that the disease progressed exponentially, more rapidly in some African countries than in others (Figure 3). The progression of the disease within the first 45 days was slowest in Nigeria compared with the other African countries. Assessing the case rate (per 100,000) during the first 45 days (or less in some African countries), the burden of COVID-19 was lowest in Nigeria (0.16 cases per 100,000 as of 11 April 2020) when compared with the selected African countries with the most cases. 

In comparison with some of the most affected countries outside Africa, the number of confirmed cases indicates that the disease spread occurred more slowly in Nigeria within the first 45 days (Figure 3). The small number of cases within the first 45 days of occurrence in the US (0.22 cases per 100,000 on 3 March 2020) and a high number of cases within the same period in Italy (29.35 cases per 100,000 on 15 March 2020) were notable. However, for these countries, the value rose to 60.89 and 244.00 cases per 100,000, respectively, as of 11 April 2020 (a month after).

**Figure 4:**
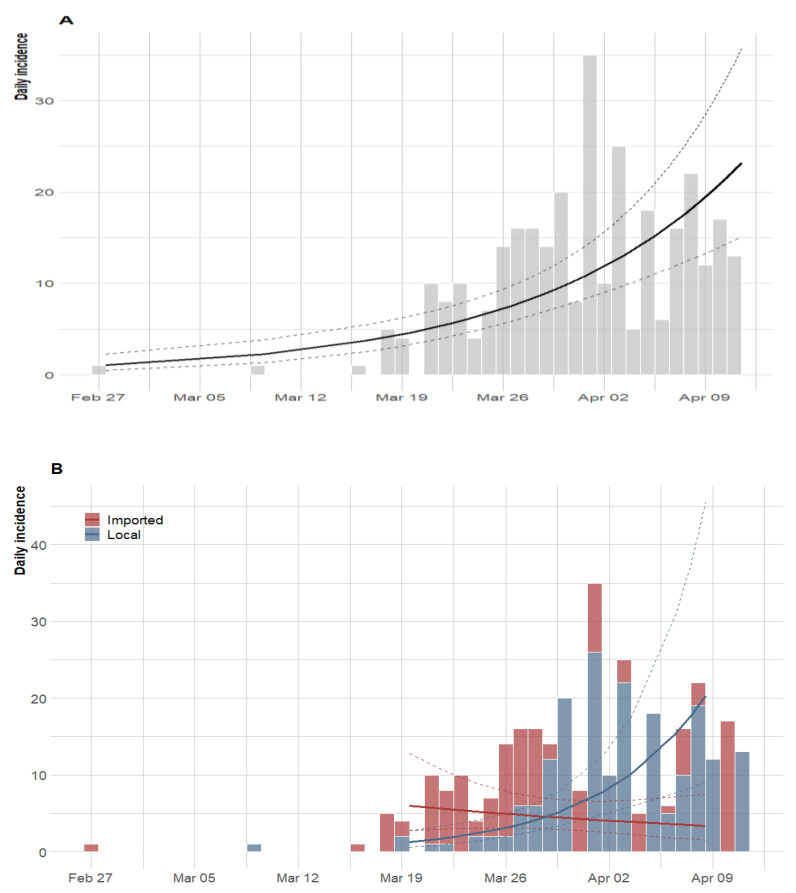
Epidemic curve of the confirmed cases of COVID-19 and the exponential growth fitting. (**A**) For all daily new cases, (**B**) disaggregated cases per transmission route as local or imported. The thick line represents the estimate surrounded by the 95% confidence interval (dashed lines).

Figure 4 presents the epidemic curves and fitted exponential growth (EG). An EG model was fitted to the overall observed incidence data (Figure 4A) and, separately for imported cases and locally transmitted incidence data (Figure 4B). As of 11 April, 2020, and at the current growth rate (0.07, 95% CI: 0.05–0.10), the doubling time of the epidemic was obtained as 9.84 days (95% CI: 7.27–15.18) based on locally transmitted incidence data only. On the other hand, when a separate model was fitted to the imported and local incidence data, the doubling time was 24.13 days (95% CI: −18.24–7.26) and 5.00 days (95% CI: 0.02–30.76), respectively. As shown in Figure 4B, the imported related fitted values show evidence of flattening but locally, transmission increases at a rate of −0.03 (95% CI: −0.10–0.04) and 0.13 (95% CI: −0.02–0.30), respectively.

**Figure 5:**
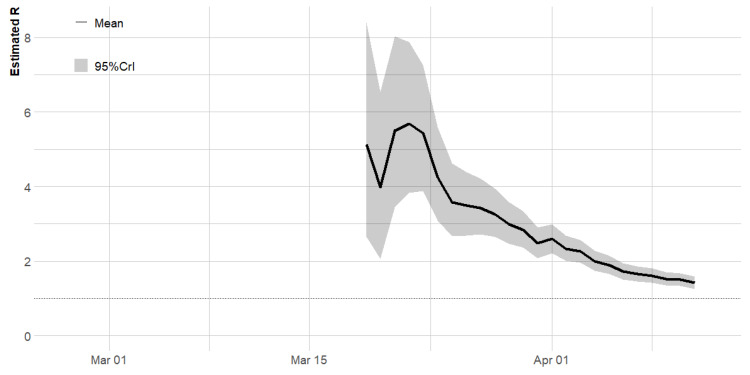
Time-varying reproduction number of COVID-19 in Nigeria based on a three-week sliding window [10,14,15] that accounted for imported and local transmission. The black line represents the posterior median, and the grey shaded region represents the 95% credible interval (CL).

Furthermore, we estimated the reproduction number for each day of the pandemic using a three-weekly window ending on day 45, while adjusting for imported cases. We used a three-week sliding window so that the number of observed cases of COVID-19 was at least 12 before estimating the reproduction number [10]. Due to the imprecise estimates at the beginning of the pandemic, the estimates of time-dependent reproduction number are not displayed in this period (Figure 5). Following Wu et al. [11], we assumed that the serial interval and the generation time have the same distribution, with a mean serial interval of 7.0 (95% CI: 5.8–8.1) days and a standard deviation of 4.5 (95% CI: 3.5–5.5). The time-varying $R_{eff}$ estimates based on these statistics are presented in Figure 5. 

The estimated reproduction number increased rapidly from a median of 4.98 (95% CrI: 2.65–8.41) at day 22 (19 March 2020), reaching a maximum value of 5.61 (95% CrI: 3.83–7.88) at day 25 (22 March 2020). The value of R decreased steadily after 20 March 2020, the day international travel ban was placed on 15 countries though it is still above the pandemic threshold of 1. The median R over the study period was 2.71, and the latest value on 11 April was 1.42 (95% CrI: 1.26–1.58). 

**Figure A3:**
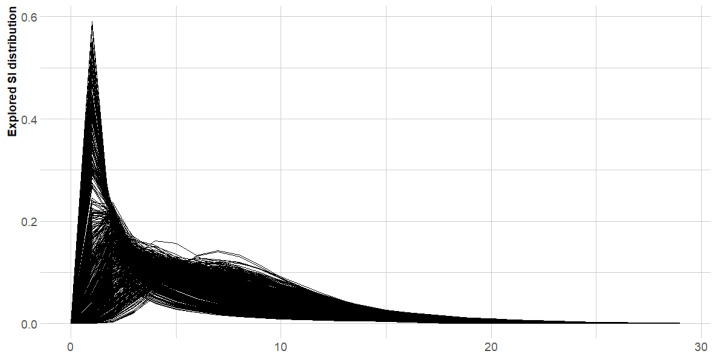
Posterior sample for serial interval distribution for time-varying reproduction number in Figure 5.

**Figure A4:**
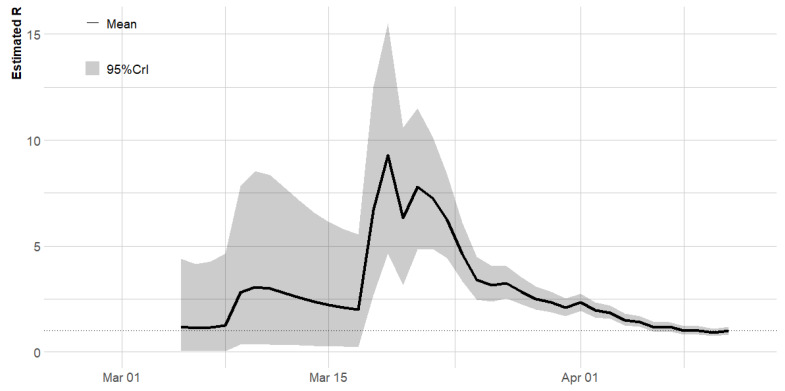
Time-varying reproduction number using one-week window.

The posterior distribution of SI based on the parametric bootstrap approach with 1000 resamples and 100 simulations is displayed in Figure A3. We assessed the changes in order to estimate the reproduction number at the early days as shown in Figure A4. Using a sliding window of one-week with a single case, the latest value at day 45 was estimated to be 0.99 (95% CrI: 0.81–1.19).

## 4. Discussion

This study is the first to provide epidemiological information on the early stages of the COVID-19 outbreak in Nigeria. Although most cases occurred in Lagos state (the economic hub of the country) and the FCT, the disease has spread to 20 states within the first 45 days. We estimated the disease growth rate and the time-varying reproduction number for the early cases of COVID-19 in Nigeria. Our modelling results show that COVID-19 was growing in the country and consequently, more cogent efforts need to be done to flatten the curve or squash the spread of the disease. The doubling time for COVID-19 importation and local transmission was 24.13 days and 5 days, respectively. This implies that based only on the importation data the epidemic will take a longer time to double but a shorter time for local transmission, which calls for additional attention. In particular, the time-varying reproduction number was above one for most of the time period, suggesting that more cases will be recorded in the country in the future. 

The first case of COVID-19 was detected in Nigeria on 27 February 2020, but the pandemic trajectory has been slow compared with other countries, and this could be attributed to early implementation of public health interventions. Among others measures, an international travel ban was imposed on 15 countries, considered to be high risks countries, on 20 March 2020 followed by a total ban of all international flights in and out of the country; early closure of all schools, universities and worship centres throughout the country and restriction on movements within and outside major cities, which were all enforced starting 29 March 2020. 

As of 11 April 2020, 318 confirmed cases were reported in the country of which about 47% were travel-related (imported) [7]. The case fatality rate was 3.14%, and 58 patients had either recovered or were in stable condition. These figures and the burden of the disease are relatively small when compared with other countries in Africa and Europe (Figure 2). However, considering the geographical landscape of Nigeria, more new cases may be confirmed in the next few weeks.

There are several reasons for the late importations of COVID-19 to Africa, other than the speculations that the disease may not be viable in temperate regions. One of the reasons is limited international travel [4]. This may be true as, economically, Nigeria is a developing country, and fewer Nigerian tourists and business personnel returning home are expected. Another reason could be the lack of exposure to the virus by Nigerian returnees. When COVID-19 emerged in China, the Chinese government introduced lockdowns with people seeking cover and sheltering in different places. Thus, Nigerian returnees might have had limited contacts in China. Hence, the first importation to Nigeria was a resident from Italy rather than from China, the original epicentre of the outbreak.

To combat emergent infectious diseases in Nigeria, the NCDC was established in 2011. Its mandate includes detecting, investigating, preventing and controlling diseases of national and international public health importance. The response of the Nigerian government to the 2014 Ebola outbreak was considered highly commendable and swift. However, the COVID-19 outbreak was quite different from that of Ebola and would, therefore, require some extra effort to handle the sudden rise in the number of outbreaks within the country. The country’s task force for the current outbreak is led by the NCDC.

An early comparison with the worse affected European countries, within the first six days, reveals a rapid acceleration of the pandemic within those countries [4], which was not the case for Nigeria and most African countries in the first 30–50 days. Consequently, Nigeria could borrow a leaf from these countries and not relent in the efforts to curb the outbreak. The setting up of nine fully functional COVID-19 laboratories across the country increased the testing capacity to 1500 a day can be considered a step in the right direction.

Nigeria, being a nation with very peculiar religious tourism and commonplace large social gatherings such as weddings and burial ceremonies, needs to enhance ‘physical’ distancing. The spread of COVID-19 has been fuelled by mass migration for religious purposes in some countries. Mass gatherings have been associated with increasing the transmission of the virus, creating high-risk conditions for the rapid global spread of infectious diseases [16]. For example, COVID-19 outbreaks were linked to several religious gathering clusters in Singapore [17,18], Malaysia [19] and South Korea [20,21,22]. It is therefore not surprising that for this year, most countries have cancelled religious activities, such as the cancellation of the Umrah pilgrimage in Saudi Arabia [23].

This study is an early investigation of COVID-19 cases in Nigeria, and as such, we acknowledge the following limitations. Firstly, though the data analysed were the official figures released by the NCDC, the actual cases in the country within the studied period could have been underreported due to the low testing capabilities. For instance, as of 6 April, 2020, the country was only able to test 5000 individuals translating to 240 per 100,000 people. Moreover, lack of proper awareness and fear of stigmatization could have hindered people with suspected cases from coming forward for testing. Secondly, since Figure 4B was based on the most plausible daily counts from the daily reports, the number of imported cases may not be in real-time, as some of the patients might have been previously included in the cases counted as missing epidemiological information. 

## 5. Conclusions

This study aimed at serving as a reminder to policymakers, health officers, disease control agencies and the general public, that although the number of confirmed cases may be relatively low in Nigeria, the risk is still very high and potentially, there could be many asymptomatic cases in the country. Thus far, the intervention in Nigeria has been timely, but the efforts need to be double up. COVID-19 cases in Nigeria are evolving in a similar way to what was observed in the early days of the pandemic in the USA. With an estimated five hospital beds per 10,000 and four medical doctors per 10,000 population (compared to US with 290 hospital beds and 25.9 medical doctors per 10,000), an outbreak of the magnitude observed in the US would be devastating. The disease is currently concentrated in Southern and North-Central Nigeria, and with ongoing Boko Haram insurgency activities in the north-east of the country, early detection and control of disease outbreaks in the north-east would be very difficult. Public gatherings/events such as religious and ceremonial gatherings need to be minimised, and restrictions on movement for an extended period. There has been no lack of knowledge of the expected economic and health costs of infectious disease outbreaks [24,25], but the country has not been adequately investing in preventive measures to mitigate the risks of large epidemic outbreaks. As the spread of the virus is likely to continue disrupting economic activities in Nigeria, the intervention measures implemented such as restrictions on movements and lockdown of cities will impose a significant economic burden and diminishing the wellbeing of people. Therefore, the government must intensity its effort in providing necessary financial incentives to the people during this period.
